# Targeting of mutant-p53 and MYC as a novel strategy to inhibit oncogenic SPAG5 activity in triple negative breast cancer

**DOI:** 10.1038/s41419-024-06987-x

**Published:** 2024-08-20

**Authors:** Valeria Canu, Sebastiano Vaccarella, Andrea Sacconi, Claudio Pulito, Frauke Goeman, Matteo Pallocca, Daniela Rutigliano, Sima Lev, Sabrina Strano, Giovanni Blandino

**Affiliations:** 1 Translational Oncology Research Unit, Department of Research, Diagnosis and Innovative Technologies, IRCCS Regina Elena National Cancer Institute, Rome, Italy; 2 Biostatistics, Bioinformatics and Clinical Trial Center, IRCCS Regina Elena National Cancer Institute, Rome, Italy; 3 Department of Research, Diagnosis and Innovative Technologies, UOSD SAFU, Translational Research Area, IRCCS Regina Elena National Cancer Institute, Rome, Italy; 4 Institute of Experimental Endocrinology and Oncology, National Research Council, Naples, Italy; 5 Department of Molecular Cell Biology, Weizmann Institute of Science, Rehovot, Israel

**Keywords:** Breast cancer, Predictive markers

## Abstract

Triple negative breast cancer (TNBC) is an aggressive disease which currently has no effective therapeutic targets and prominent biomarkers. The Sperm Associated antigen 5 (SPAG5) is a mitotic spindle associated protein with oncogenic function in several human cancers. In TNBC, increased SPAG5 expression has been associated with tumor progression, chemoresistance, relapse, and poor clinical outcome. Here we show that high SPAG5 expression in TNBC is regulated by coordinated activity of YAP, mutant p53 and MYC. Depletion of YAP or mutant p53 proteins reduced SPAG5 expression and the recruitment of MYC onto SPAG5 promoter. Targeting of MYC also reduced SPAG5 expression and concomitantly tumorigenicity of TNBC cells. These effects of MYC targeting were synergized with cytotoxic chemotherapy and markedly reduced TNBC oncogenicity in SPAG5-expression dependent manner. These results suggest that mutant p53-MYC-SPAG5 expression can be considered as bona fide predictors of patient’s outcome, and reliable biomarkers for effective anticancer therapies.

## Introduction

Breast cancer (BC) is the most frequent cancer among women worldwide. Current histopathological classification and targeted treatment significantly improved prognosis of luminal and HER-2 enriched tumors, whereas triple negative breast cancer (TNBC), routinely classified as a group of highly heterogenous disease, still lacks of specific prognostic and therapeutic biomarkers [1]. Beyond transcriptomic classification of TNBC into Basal-like 1/2, mesenchymal, luminal and androgen receptor positive subtype, further stratification is critical to dissect their genetical landscape and plan effective anticancer therapy [2–4].

Sperm associated Antigen 5 (SPAG5) is an essential component of mitotic spindle, needed for chromosome alignment and sister chromatids segregation during anaphase [5–7]. Frequently overexpressed in advanced BC [8], including TNBC, SPAG5 is considered to be an efficient prognostic factor [9, 10]. Amplification of *SPAG5* gene is associated with BC growth, positive lymph node status, anthracycline vulnerability, poor prognosis, and TP53 mutations [11]. *TP53* is the most common mutated gene across cancers, including BC. *TP53* gene, encode for a transcription factor involved in cell cycle regulation, apoptosis, DNA repair, and metabolic processes [12].

In BC, TP53 mutations account for about 30% but their frequency markedly increases to ~80% in TNBC [6]. Major alterations in the *TP53* gene are missense mutations, which are frequently located in the DNA binding domain (R175, R248, R249, R273, R282), and result in gain of oncogenic activity [13]. In cancer cells, the oncogenic function of mutant p53 proteins (mut-p53) can be enhanced through interaction with other transcription factors or coactivator, including the Hippo pathway transducer YAP [14, 15].

Previously, we showed that SPAG5 depletion strongly impaired proliferation and migration of TNBC cell lines, and identified SPAG5 as a direct transcriptional target of YAP/TAZ/TEAD [16]. Here, we analyzed the expression levels of SPAG5, YAP and p53 transcripts in METABRIC dataset, and found that the expression levels of YAP and SPAG5 increased along with p53 mutations in more aggressive BC histotypes. This, was associated with elevated level of TP53 mutant-dependent MYC signature. MYC depletion, or inhibition by small-molecule inhibitors, reduced SPAG5 expression, clonogenicity, and sensitized TNBC cells lines to cisplatin, 5-fluorouracil and paclitaxel. We further found that SPAG5 is a direct target of MYC, and collectively showed that mut-p53, YAP and MYC coordinately function to regulate SPAG5 expression. Our data, introduce mut-p53-MYC-SPAG5 expression as bona fide predictors of patient’s outcome, and reliable biomarkers for effective anticancer therapies.

## Materials and methods

### Cell lines and culture conditions

All cell lines were obtained from ATCC. Human cell line MDA-MB-231 was maintained in Dulbecco’s Modified Eagle Medium (DMEM) (Invitrogen-GIBCO, Carlsbad, CA, USA) supplemented with 10% Fetal Bovine Serum (FBS) (Invitrogen-GIBCO), 100 U/ml penicillin and 100 μg/ml streptomycin. Human cell line MDA-MB-468 was maintained in DMEM High Glucose (EUROCLONE) containing 10% inactivated FBS (Invitrogen-GIBCO), 2mM l-glutamine, 100 U/ml penicillin and 100 μg/ml streptomycin. Human cell line SUM-159PT was maintained as DMEM/F-12.GLUTAMAX (Invitrogen-GIBCO, Carlsbad, CA, USA) supplemented with 10% FBS (Invitrogen-GIBCO), 10 μg/ml Insulin, 100 U/ml penicillin and 100 μg/ml streptomycin. Human cell line SKBR-3 was maintained in RPMI medium (Invitrogen-GIBCO, Carlsbad, CA, USA) supplemented with 10% inactivated FBS (Invitrogen-GIBCO), 100 U/ml penicillin and 100 μg/ml streptomycin. All cells were cultured at 37 °C in 5% CO2.

### Establishment of organoids culture

Tissues were collected in MACS Tissue Storage Solution (130-100-008, Miltenyi Biotec) supplemented with 100 U/ml penicillin, 100 mg/ml streptomycin and 100 mg/ml antimycotic. After washing in PBS 1×, samples were mechanically minced. Single-cell suspensions was obtained using Tumor Dissociation Kit (130-095-929, Miltenyi Biotec), according to the manufacturer’s instructions. Pellet was resuspended in 30 μl of Matrigel (Corning) and plated in a prewarmed 24-well plate and placed in 5% CO_2_ incubator for 30 min to allow Matrigel polymerization. Each well was then covered with organoids expansion medium (1× B27 supplement, 5 mM Nicotinamide, 1.25 mM N-Acetylcystein, 250 ng/mL R-spondin 1, 5 nM Heregulin β-1, 100 ng/ml Noggin, 20 ng/ml FGF-10, 5 ng/ml FGF-7, 5 ng/ml EGF, 500 nM A83-01, and 500 nM SB202190).

The organoids were passaged every 1–2 weeks, depending on proliferation rate [17].

Bright-field imaging of organoids was performed on an NEXCOPE microscope.

### Plasmids and siRNA transfections

MDA-MB-231, MDA-MB-468, SUM-159PT and SKBR-3 human cells lines were transfected for 48 h, using the following siRNAs: si-p53 5′-GACUCCAGUGGUAAUCUAC-3′, si-YAP 5′-GACAUCUUCUGGUCAGAGA-3’ and si-MYC 5′-GGACUAUCCUGCUGCCAAG-3′ at a final concentration of 100 pM using *Lipofectamine RNAi* MAX (Invitrogen) according to the manufacturer’s instructions. si-GFP 5′-UUCAGCGUGUCCGGGGAG-3′ were used as controls. For stable overexpression of SPAG5, MDA-MB-231 cells were transfected with a plasmid pCMV6, containing the complete open-reading frame of the human SPAG5 transcript driven by the CMV promoter (OriGene Technologies) using *Lipofectamine 3000* (Invitrogen) according to the manufacturer’s instructions.

### cDNA synthesis and RT-qPCR

One microgram of total RNA was reverse-transcribed at 37 °C for 60 min in the presence of random hexamers and Moloney murine leukemia virus reverse transcriptase (Invitrogen). Specific oligonucleotide primers for GAPDH FW: 5′-GAGTCAACGGATTTGGTCGT-3′ RV: 5′-GACAAGCTTCCCGTTCTCAG-3′ SPAG5 FW: 5′-ACTGAGAGTGATGTTCCTGGA-3′ RV: 5′-CTAACTCCTTGTCAGAGCGC-3′, TP53 FW: 5′-GTCTGGGCTTCTTGCATTCT-3′; RV: 5′-AATCAACCCACAGCTGCAC-3′; MYC FW: 5′-CTCCTGGCAAAAGGTCAGAG-3′, RV: 5′-TCGGTTGTTGCTGATCTGTC-3′ MYC/SPAG5 promoter (Region 2171) FW: 5’: AGTCAACACCAATCCTATACTCC-3′, RV: 5′-AGTGCTAGGATTACAGACGTG; MYC/SPAG5 promoter (Region 908) FW: 5′-ATCCTAGCACTTTGGGAGGC-3′, RV: 5′- TTCAAGCGATTCTCCCACCT-3′, were used for PCR analyses. Gene expression levels were measured by quantitative real-time PCR according to the manufacturer’s instructions.

### Lysate preparation and immunoblotting analyses

Cells were lysed in buffer with 50 mM Tris–HCl pH 8, with 1% NP-40 (IgepalAC-630), 150 mM NaCl, 5 mM EDTA, and fresh protease inhibitors. Extracts were centrifuged at 14,000 rpm for 10 min to remove cell debris. Protein concentrations were determined by colorimetric assay (Bio-Rad). Western blotting was performed using the following primary antibodies: rabbit polyclonal anti-SPAG5 antibody (60940, Cell Signaling), which detects both isoforms of the SPAG5 protein [18], rabbit polyclonal anti-YAP PA1 (46189, Invitrogen), rabbit monoclonal anti-cyclin B1 (ab215436, Abcam), rabbit monoclonal anti MYC-p(Ser62) (13748, Cell Signaling), mouse monoclonal anti-p53 (DO-1) (sc-126, Santa Cruz Biotechnology), rabbit monoclonal anti-cleaved PARP (Cell Signaling), rabbit monoclonal anti-p21 (2947, Cell Signaling), mouse monoclonal anti-c-MYC (sc-40, Santa Cruz Biotechnology), mouse monoclonal anti-tubulin (ab56676, Abcam), mouse monoclonal anti-GAPDH (sc-47724, Santa Cruz), mouse monoclonal anti-Nucleolin (sc-803, Santa Cruz), mouse monoclonal anti-Actin (sc-47778, Santa Cruz). Secondary antibodies used were goat anti-mouse, goat anti-rabbit conjugated to horseradish peroxidase (Amersham Biosciences, Piscataway, NJ, USA). Immunosignal were detected by the chemiluminescent method (Uvitec Alliance, Cambridge).

### Immunofluorescence

For immunofluorescence assay, 4 × 10^4^ cells/well were seeded in culture media onto glass coverslips (Paul Marienfeld, Lauda-Königshofen, Germany) in 6-well dishes (Corning Inc.). Cells were fixed with 4% formaldehyde in PBS for 15 minutes at room temperature and then permeabilized with 0.25% Triton X-100 in PBS for 10 minutes. After blocking with 5% BSA/PBS at room temperature slides were incubated in 5% BSA/PBS overnight at +4° with Rabbit anti-SPAG5 antibody A301-512A Bethyl Laboratories, (Montgomery, USA), mouse monoclonal anti-YAP (SC-101199 Santa Cruz Biotechnology), mouse monoclonal anti-c-MYC (sc-40, Santa Cruz Biotechnology), mouse monoclonal anti-p53 (DO-1) (sc-126, Santa Cruz Biotechnology), according to the manufacturer’s instructions. Secondary antibody used were Alexa Fluor 594 (1:500; Thermo Fisher Scientific). Nuclei were stained with DAPI (Thermo Fisher Scientific).

### Cell treatment

In total, 10^5^ BC cell lines were plated in 60-mm dishes. After 24 h cells were treated with the following drugs all were purchased from Selleck Chemicals: Dasatinib (S5254), JQ-1 (S7110), CPI-0610 (S7853), INCB054329 (S8753), OTX-015 (S7360), Cisplatin (S1166), Paclitaxel (S1150), 5-Fluorouracil (S1209), Doxorubicin (S1208), Irinotecan (S1198), Vinorelbine ditartrate (S4269) at the specified final concentration.

### Colony-formation assay

BC cell lines were grown in 60–80% confluence and transfected with siRNAs using *Lipofectamine RNAi* MAX, according to the manufacturer’s instructions, or treated with the indicated compounds. After 48 h, 500 cells were seeded in six-well dishes (Corning-Costar, Tewksbury, MA, USA). Fresh media (25%) was added every 3 days. After 7–10 days of culture, the colonies were stained with crystal violet and counted.

### Cell viability assay

In all, 8 × 10^2^ cells were seeded into 96-well plates. Cell viability was assessed using ATPlite assay (Revvity, Massachusetts, USA), according to the manufacturer’s instructions. Combination Index (CI) was calculated by calcusyn software according to the manufacturer’s instructions.

### PDTO morphological measurement

The measurement of the perimeter, width, length, and area dimensions of organoids was assessed using the Opera Phenix® Plus high-throughput microplate confocal imager (Revvity, Massachusetts, USA) and calculated using Harmony High-Content Imaging and Analysis Software [19].

### Promoter analysis

FASTA sequences of human SPAG5 (NM 06461) promoter (5000 bp upstream of the TSS) were downloaded from UCSC Genome Browser on-line database. LASAGNA-Search 2.0 were used to identify predicted transcription factor binding sites. TRANSFAC matrices were used for the analysis.

### ChIP experiments

Chromatin immunoprecipitation was performed as previously reported [16]. The chromatin solution was immunoprecipitated with rabbit monoclonal anti-MYC-p(Ser62) (Cell Signaling) or rabbit polyclonal anti-H4Ac (2594, Cell Signaling). The immunoprecipitations were performed using Pierce ChIP-grade Protein G magnetic beads (Thermo Fisher Scientific). The immunoprecipitated and purified chromatin was subjected to RT-qPCR. The promoter occupancy was analyzed by RT-qPCR using the SYBR Green assay (Applied Biosystems). Normalization was performed to the amount of input chromatin.

### Statistical analysis

Normalized gene expression of BC patients was obtained from Metabric dataset and Broad Institute TCGA Genome Data Analysis Center (2016); TCGA data from Broad GDAC Firehose 2016_01_28 run. Broad Institute of MIT and Harvard. Dataset. 10.7908/C11G0KM9.

DEseq2 pipeline was used for pre-process analysis.

Unsupervised hierarchical clustering was performed to identify specific patterns of gene expression using the Euclidean distance metric and average linkage.

Survival and progression-free survival analyses were conducted using the Kaplan–Meier method. To determine the statistical significance of differences between survival curves, a log-rank test was applied. High and low expression values for subgroups of patients were assessed by calculating z-scores of a single gene or by the z-scores of the mean value of a signature of genes.

The impact of clinical variables on survival curves was investigated using a multivariate Cox proportional hazard regression model.

Statistical significance for the modulation of a single gene among patient subgroups was inferred using the Student’s t-test when comparing two groups or the ANOVA test when comparing more than two groups.

### Pathway analysis

A Gene Set Enrichment Analysis (GSEA) was conducted using the GSEA software available at https://www.gsea-msigdb.org/gsea/index.jsp. This analysis utilized curated gene sets from the Molecular Signature Database (MSigDB) derived from KEGG, Hallmark, and Reactome collections. The GSEA was run in preranked mode using the classic metric, and 1000 permutations were performed to assess the statistical significance of pathway enrichment. Genes were ranked based on the formula SCORE = sign (FC) * (-log10Pvalue), which combined fold change (FC) and statistical significance (*P*-value) information. The above analyses were performed using MATLAB R2022a software.

## Results

### Expression levels of SPAG5, YAP and mut-p53 in BC subtypes

Recently, we showed that in BC SPAG5 expression is transcriptionally sustained by YAP-TAZ-TEAD interaction [16], while previous reports suggest that mut-p53 proteins frequently crosstalk with YAP or other determinants of the Hippo signalling pathway [14]. We therefore investigated whether YAP, mut-p53 and SPAG5 were mutually aberrantly expressed in BCs patients.

**Fig. 1:**
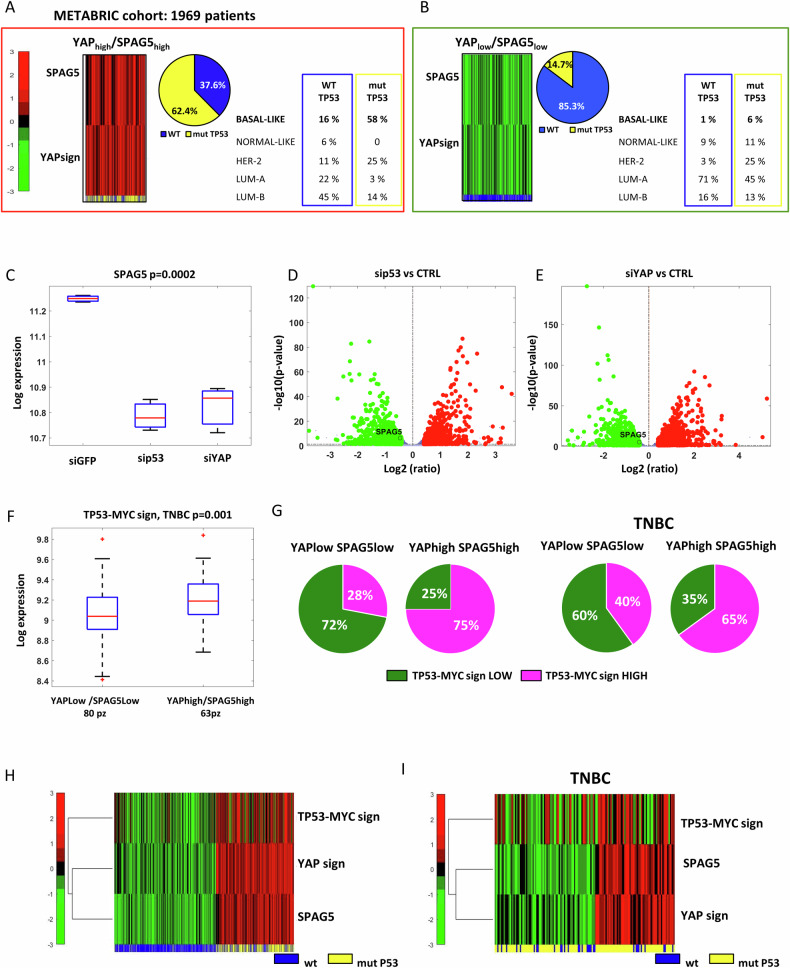
Expression levels of SPAG5, YAP and mutant P53 and their correlation to a p53-dependent MYC signature in breast cancer patients. **A**, **B** Heatmaps (on the left) of normalized expression levels of SPAG5, YAP and p53 status in METABRIC dataset. Pie-charts (in the middle) frequencies of p53 mutations in breast cancer patients from METABRIC dataset stratified for combined expression of SPAG5 and YAP. Tables (on the right) breast cancer histotype classification in the different group of breast cancer patients stratified for combined expression of TP53, SPAG5 and YAP. **C** Box-plot of SPAG5 expression in MDA-MB-468 breast cancer cells 48 h post sip53, siYAP or siCTRL transfection. siRNAs transfection assessed by RNA sequencing analysis. Volcano plots of all modulated transcripts as assessed by RNA sequencing of sip53 (**D**) or siYAP (**E**) transfected cells. Red dots mark upregulated and green dots downregulated genes. **F** Box-plot of expression levels of TP53-MYC-target genes signature in breast cancer patients from METABRIC dataset, stratified for different combined levels of SPAG5 and YAP expression signatures. **G** Pie-charts represent the frequencies of TP53-MYC-target genes signature expression in breast cancer patients (left panel) and in TNBC patients (right panel) from METABRIC dataset, stratified for different combined levels of SPAG5 and YAP signatures. Heatmaps of normalized expression levels of SPAG5, YAP, TP53-MYC-target genes signature, and p53 in breast cancer patients (**H**) and in TNBC patients (**I**) from METABRIC dataset.

We classified 1969 BC patients from METABRIC dataset, containing clinical and genomic data from primary BC, into four different groups, based on the expression levels of SPAG5 and YAP signatures: SPAG5^high^/YAP^high^, SPAG5^low^/YAP^low^, SPAG5^high^/YAP^low^_,_ SPAG5^low^/YAP^high^_,_ (Heatmap Fig. 1A, B, Fig. S1A, B). For each group, we analysed TP53 status wild-type (wt-TP53) or mutated, and the prevalence of BCs histotypes (Normal-like, Basal-like, HER-2, luminal-A, luminal-B) as a measurement of tumor malignancies (Pie-charts Fig. 1A, B, Fig. S1A, B). Figure 1A depicts patients characterized by SPAG5^high^ and YAP^high^: in this group higher frequency of mut-TP53 (62.4%) was associated with a higher risk to develop more aggressive tumor histotype (Basal-like = 58%). In contrast, patients carrying wt-p53 protein with SPAG5^high^ and YAP^high^, mainly developed luminal features, while the frequency of Basal-like histotype decreased to 16% (Table in Fig. 1A). Opposite scenario was observed by analysing patients with SPAG5^low^ and YAP^low^. In this group, TP53 mutations were less prevalent compared to the other categories (14.7%) and the incidence of basal-like subtype was decreased to 6% (Fig. 1B). We also considered patients with SPAG5^low^/YAP^high^ or SPAG5^high^/YAP^low^. Beyond similar frequencies of TP53 mutations 28.5% in SPAG5^high^/YAP^low^ and 33% in SPAG5^low^/YAP^high^ groups, 41% of patients with YAP^high^ and mut-p53 were associated with basal-like subtype (Fig. 1SA), while SPAG5 overexpression act as a self-sufficient supporter of tumor growth. Of interest, normal-like phenotype shows the lower prevalence when SPAG5 is highly expressed (Fig. 1SB). Collectively, these observations suggest that in BC patients, SPAG5, YAP and mut-p53 were concomitantly expressed and clinically associated with tumor aggressiveness.

### YAP and mut-p53 regulate SPAG5 expression independently

Previous studies have shown that mut-p53 and YAP recruit transcription factors to the promoters of shared target genes, supporting cell cycle progression and tumor growth [14, 20]. RNA-sequencing of MDA-MB-468 TNBC cells depleted of mutant TP53 (R273H) or YAP genes, showed reduced SPAG5 mRNA expression in these cells (Fig. 1D, E), suggesting that both YAP and mut-p53 regulate SPAG5 expression. Consistent with earlier reports [20], GSEA and KEGG pathway analysis identified cell cycle and mitotic checkpoint pathways regulated by mut-p53 and YAP. Among the negatively enriched pathways in TP53-depleted cells, we focus on MYC targets, because of its central oncogenic signal in BC and its compelling role in anticancer target therapy (Fig. S1C). Furthermore, concomitant high expression of mut-p53 and MYC is associated with poor prognosis in TNBC patients [21–24].

### SPAG5, mut-p53, YAP and TP53-MYC-target gene signature are prognostically relevant for TNBC

Normalized high expression of SPAG5 and YAP were significantly associated with high levels of a TP53-dependent MYC-target gene signature (TP53-MYC target signature) in all BC (p = 5.1277 e-^77^) and in TNBC patients (p = 0.001) (Figs. 1F, S1E) [24]. Notably, 75% of all BC and 65% of TNBC patients with SPAG5^high^ and YAP^high^ were also highly enriched in TP53-MYC target signature (Fig. 1G). Furthermore, TP53 mutations prevailed within patients with high levels of SPAG5, YAP and TP53-MYC signature (Fig. 1H, I). COX multivariate analysis adjusted for tumoral stage, nodal stage, histotype, age and menopausal state, further predicts that SPAG5, mut-p53 and YAP are prognostic determinants for BC, independently or in association with TP53-MYC signature (Table 1). Collectively, these findings reveal that expression of SPAG5, YAP, mut-p53 and TP53-MYC signature, are mutually connected and significantly prognostic for BC patients.

**Table 1:** COX multivariate analysis adjusted for T, N, stage and histotype, age and menopausal state, of SPAG5, mutant p53, YAP and TP53-MYC signature expression in breast cancer patients from METABRIC dataset.

	YAPsign		SPAG5		MYCsign		MUT-TP53	
MULTIVARIATE model	HR [CI95%]	*p*	HR [CI95%]	*p*	HR [CI95%]	*p*	HR [CI95%]	*p*
YAPsign + SPAG5	1.2479 [1.1531–1.3506]	4.02638E−08	1.2028 [1.119–1.2929]	5.36966E−07				
YAPsign + SPAG5 + MYCsign	1.2018 [1.1064–1.3054]	1.32534E−05	1.1383 [1.0492–1.2349]	0.001834135	1.1493 [1.0536–1.2537]	0.001707822		
YAPsign + SPAG5 + MYCsign+TP53	1.1454 [1.0514–1.2479]	0.001898846	1.1067 [1.0182–1.2029]	0.017148844	1.1027 [1.0095–1.2044]	0.030034473	1.4603 [1.2201–1.7479]	3.64752E−05

### Mut-p53 depletion reduces TP53-MYC target signature and SPAG5 expression in BC cell lines

**Fig. 2:**
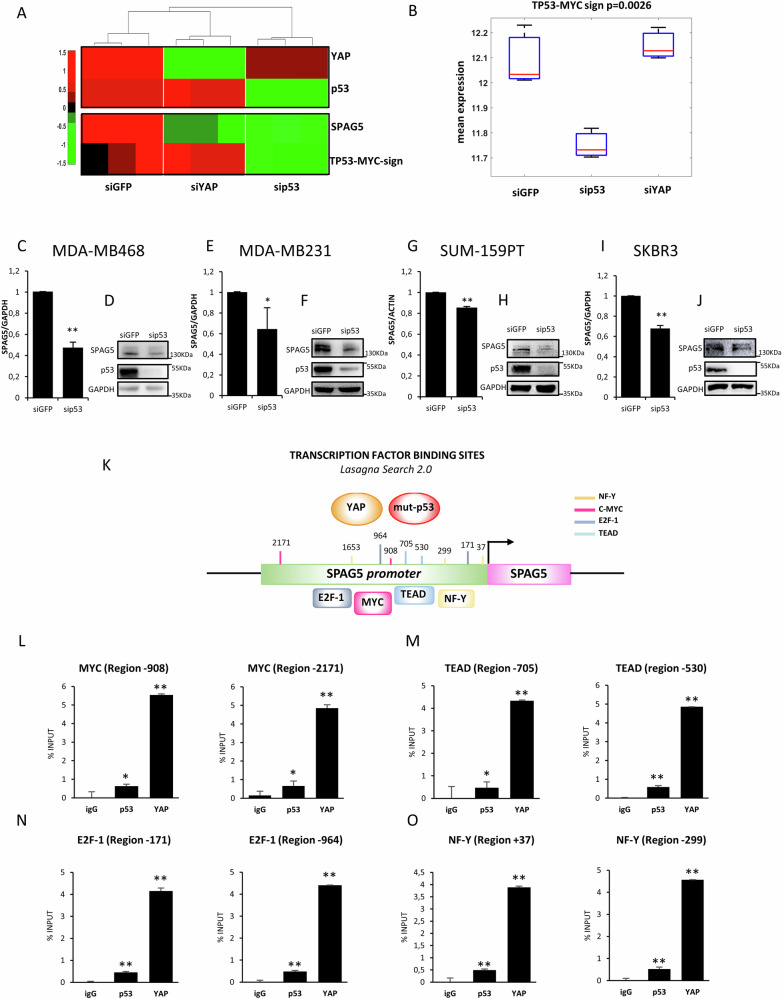
Recruitment of mutant P53 and YAP to SPAG5 promoter. **A** Heatmap normalized expression of SPAG5 and TP53-MYC-target genes signature in MDA-MB-468 breast cancer cell line was determined by RNA sequencing analysis 48 hr post transfection with sip53, siYAP or siGFP. **B** Box-plot of TP53-MYC-target genes signature expression in MDA-MB-468 cell line 48 hr post transfection with sip53, siYAP and siGFP. (**C**, **E**, **G–I)** qPCR the expression levels of SPAG5 in MDA-MB-468 (**C**), MDA-MB-231 (**E**), SUM-159PT (**G**), and SKBR3 (**I**), 48 h post transfection with siGFP and siP53 transfection was assessed by quantitative PCR. **D-F-H-J** Western blot analysis of SPAG5 protein level in MDA-MB-468 (**D**), MDA-MB-231 (**F**), SUM-159PT (**H**), and SKBR3 (**J**), 48 h post transfection with siGFP and siP53. **M** Schematic representation of SPAG5 promoter with c-MYC, NF-Y, E2F-1, and TEAD, putative binding sites predicted by LASAGNA Search 2.0. Chip analysis of p53 and YAP enrichment on transcriptional binding sites of MYC-p(Ser62) (**L**), TEAD (**M**), E2F-1(**N**) and NF-Y (**O**) onto SPAG5 promoter. (*P value < 0.05; **P value < 0.001).

Mut-p53 depletion in MDA-MB-468 cells reduced the expression of TP53-MYC target signature (Fig. 2A, B), and significantly impaired the expression of G3BP1, XRCC6 and CCT3, TP53-dependent MYC target genes, as evaluated by qRT-PCR (Fig. S2A) [24]; concomitant decrease of SPAG5 expression (Fig. 2A, B), strongly suggests a common transcriptional program. To further assess the impact of mut-p53 on SPAG5 expression, we knocked it down in four BC cell lines harboring different TP53 missense mutations: MDA-MB-468 (R273H), MDA-MB-231(R280K), SUM-159 (R158L) TNBC cell lines, and SKBR3 (R175H) HER-2 positive BC cells. Intriguingly, we found that SPAG5 transcript (Fig. 2C, E, G, I) and protein (Fig. 2D, F, H, J) levels were significantly reduced in all p53-depleted cells, highlighting the impact of mut-p53 on SPAG5 expression.

### Mut-p53 and YAP recruit transcription factors to SPAG5 promoter

We showed that YAP depletion reduced SPAG5 expression (Figs. 2A, S2B) [16]. Chip-Seq data on MDA-MB-231 cells, revealed that mut-p53 and YAP colocalize with c-MYC, E2F1, and TEAD4 binding sites on SPAG5 promoter (www.cistrome.org/db) (Fig. S2C). Furthermore, LASAGNA algorithm (Length Aware Alignment Guided by Nucleotide Association) predicted specific binding sites of cMYC, NF-Y, E2F and TEAD on SPAG5 promoter (Fig. 2K). We validated the *in-silico* analysis by ChIP-PCR on MDA-MB-468 TNBC cell line. Rousingly, we found a remarkable increase in the recruitment of mut-p53 and YAP to the SPAG5 promoter within c-MYC, E2F1, NF-Y, and TEAD4 binding sites, highlighting novel transcriptional regulators of SPAG5 expression (Fig. 2L–O).

### c-MYC depletion reduces SPAG5 expression and clonogenic ability of TNBC cell lines

**Fig. 3:**
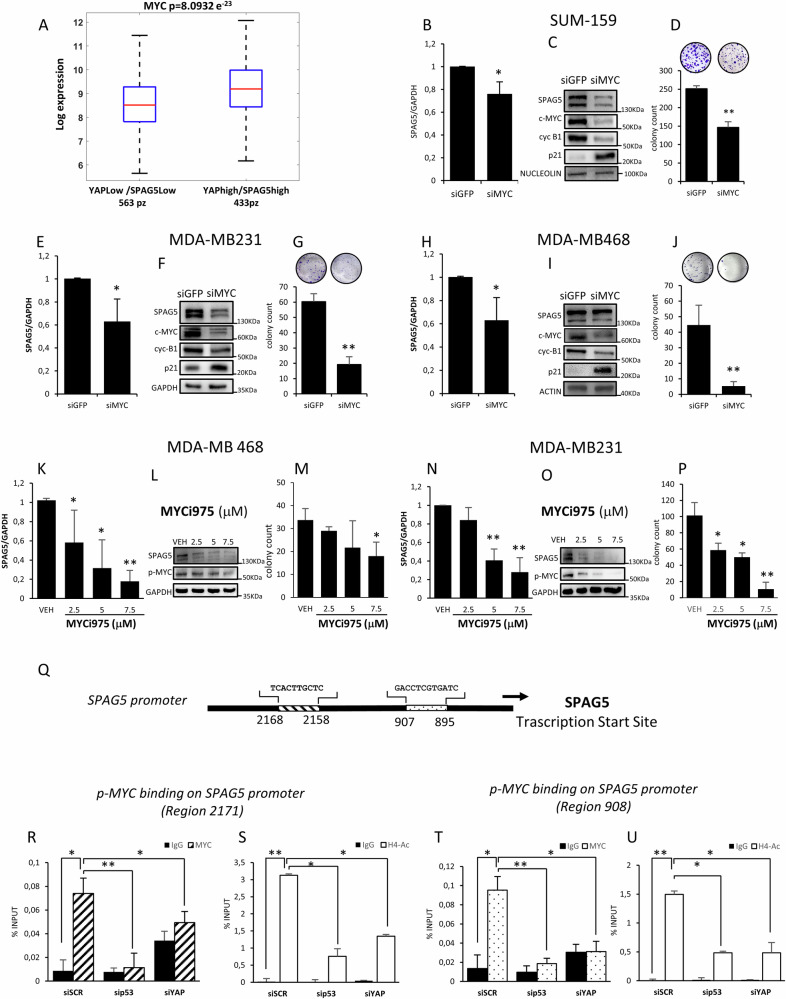
MYC directly binds SPAG5 promoter. **A**, **B** Box-plots expression levels of c-MYC in breast cancer patients (A) from METABRIC dataset, stratified for different combined levels of SPAG5 and YAP signature expression. qPCR analysis of SPAG5 expression levels in SUM-159 (**B**), MDA-MB-231 (**E**) and MDA-MB-468 (**H**) 48 h post transfection with siGFP and siMYC. Western blot analysis shows the protein levels of SPAG5, c-MYC, cyclin B1 and p21 protein levels of whole-cell lysate of SUM-159 (**C**), MDA-MB-231 (**F**) and MDA-MB-468 (**I**) 48 hr post transfection with siGFP and siMYC. **D**, **G**, **H** Clonogenic assay. Representative micrographs of colonies formed by SUM-159 (**D**), MDA-MB-231 (**G**) and MDA-MB-468 (**J**) cells transfected with siGFP and siMYC. After 7–10 days, colonies were stained with crystal violet and counted. Column graphs show colonies number and *P* value from three independent experiments. **K–N** qPCR analysis of SPAG5 expression level in MDA-MB-468 (**K**) and MDA-MB-231 (**N**) assessed by quantitative PCR 48 hr post treatment with 2.5 μM −5 μM −7.5 μM of MYCi975. **L–O** Western blot analysis of SPAG5 and p-MYC protein levels in whole-cell lysate of MDA-MB-468 (**L**) and MDA-MB-231 (**O**) cells 48 hr post treatment with 2.5 μM −5 μM −7.5 μM of MYCi975. **M–P** Clonogenic assay. Representative micrographs of colonies formed by MDA-MB-468 (**M**), MDA-MB-231 (**P**) treated for 48 hr with 2.5 μM −5 μM −7.5 μM of MYCi975, before seeding at clonal density. After 7–10 days, colonies were stained with crystal violet and counted. Column graphs show colony count and *P* value from three independent experiments. **Q** Schematic representation of the SPAG5 promoter with the putative MYC-binding sites as predicted by LASAGNA. Search 2.0. **R–T** ChIP analysis of the MYC-p(Ser62) binding on SPAG5 promoter in MDA-MB-468 cells after TP53 and YAP interference, detected by RT-qPCR analysis. **S–U** Transcriptional active chromatin evidenced by anti-H4-Acetylate antibody. Data shown as one of three independent replicates. (*P value < 0.05; **P value < 0.001).

To further explore the relation between SPAG5 and MYC, we assessed their association in METABRIC dataset. As shown, high levels of SPAG5 and YAP were significantly associated with c-MYC expression in BCs patients (p = 8.0932 e-23) (Fig. 3A). A similar trend was observed in TNBC patients, although it was not statistically significant (p = 0.12) (Fig. S3A). Therefore, we knocked down c-MYC in MDA-MB-468, MDA-MB-231 and SUM-159 TNBC cell lines. SPAG5 mRNA (Fig. 3B, E, H) and protein (Fig. 3C, F, I) levels were markedly reduced in MYC-depleted cells. Expression of NME1 G3BP1, XRCC6, CCT3 and TCP1 [24], were decreased (Fig. S3B), and the number of the colonies formed by TNBC cell line were also significantly reduced (Fig. 3D, G, J). These results, together with the downregulation of SPAG5 and cyclin-B1, and upregulation of CDK inhibitor p21 proteins, suggest that depletion of c-MYC reduced SPAG5 expression and consequently BC cell proliferation (Fig. 3C, F, I). To corroborate these findings, we used the small molecule MYC-inhibitor, MYCi975 [25]. Noticeably, MYCi975 induced a dose dependent downregulation of SPAG5 mRNA and protein levels, (Figs. 3K, L, N, O, S3C, D), reduced expression of MYC-p(Ser62), G3BP1, XRCC6, NME1 and TCP1 (Figs. 3L, O, S3D, F, G), and the number of colonies formed by in MDA-MB-468, MDA-MB-231 and SUM-159 cells (Figs. 3M, P, S3E).

### SPAG5 is a direct transcriptional target of MYC

Two different c-MYC binding sites were predicted by Lasagna 2.0 algorithm on SPAG5 promoter (Fig. 3Q). ChIP-PCR assay demonstrated a significant enrichment of active MYC-p(Ser62) on the selected regions of SPAG5 promoter (Fig. 3R, T), concomitantly with increased Histone H4 acetylation, marker of transcriptional active chromatin (Fig. 3S, U). Furthermore, p53 or YAP depletion (Fig. S3H), markedly reduced MYC-p(Ser62) recruitment on SPAG5 promoter and histone acetylation on the selected regulatory regions (Fig. 3R–U). SF3B3, MYC-target gene, was used as a positive control (Fig. S3J) [24]. Collectively these findings show that SPAG5 is a direct transcriptional target of MYC, p53 and YAP axis.

### JQ-1 and Dasatinib inhibit SPAG5 expression troughs MYC

**Fig. 4:**
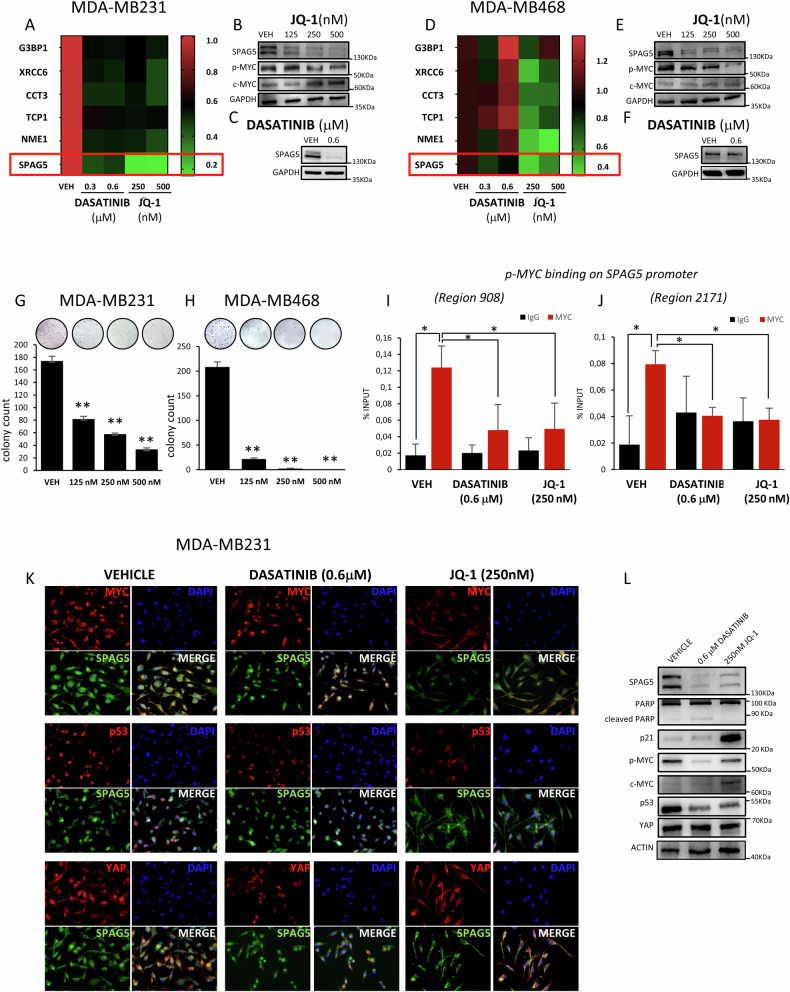
Pharmacological targeting of MYC impairs SPAG5 expression. **A**–**D Heatmaps** show the expression levels of SPAG5, G3BP1, XRCC6, CCT3, TCP1 and NME1 in MDA-MB-231 (**A**) and MDA-MB-468 (**D**) TNBC cell lines, 48 h post treatment with 0.3–0.6 μM of DASATINIB or 72 h post treatment with 250–500 nM of JQ-1. Western blot analysis shows the protein levels of SPAG5, MYC-p(Ser62), and c-MYC in whole-cell lysate of MDA-MB-231 (**B**, **C**) and MDA-MB-468 (**E**, **F**) 72 h post treatment with 125–250–500 nM of JQ-1 (**B**–**E**) or 48 hr post treatment with 0.6 μM of DASATINIB (**C**–**F**). **G**, **H** Clonogenic assay. Representative micrographs of colonies of MDA-MB-231 (**G**) or MDA-MB-468 (**H**) cells treated for 72 hr with 125–250–500 nM of JQ-1 before seeding at clonal density. After 7–10 days, colonies were stained with crystal violet and counted. Column graphs show colony count and *P* value from three independent experiments. **I**, **J** ChIP analysis of the MYC-p(Ser62) binding on SPAG5 promoter in MDA-MB-231 cell line after 0.6 μM of DASATINIB and 250 nM of JQ-1 treatments, detected by RT-qPCR analysis. Transcriptional active chromatin is evidenced by anti-H4-Acetylate antibody. **K** Immunofluorescent assay. Representative images of MDA-MB-231 cells treated with either 0.6 μM DASATINIB or 250 nM JQ-1 for 72 hr, and immunostained with anti-SPAG5, anti-MYC, anti-YAP, and anti-p53 antibodies. Nuclei were stained with DAPI. Scale-bar, 20 μM. **L** Western blot shows the protein levels of SPAG5, PARP, cleaved PARP, p21, MYC-p(Ser62), c-MYC, TP53 and YAP in whole-cell lysate of MDA-MB-231 cell line 48 h post treatment with 0.6 μM of DASATINIB and 72 h of 250 nM of JQ-1 treatments. (*P value < 0.05; **P value < 0.001).

Numerous studies have shown that BET inhibitor JQ-1 downregulates MYC transcription and target genes expression [26]. Therefore, we examined the ability of JQ-1 to modulate SPAG5 expression and colony formation of TNBC cell lines. As shown, JQ1 markedly reduced SPAG5 transcript and protein levels (Figs. 4A, B, D, E, S4A, B), NME1 G3BP1, XRCC6, CCT3, TCP1 genes expression (Figs. 4A, D, S4A) and the number of the colonies formed by MDA-MB-231, MDA-MB-468 and SUM-159 TNBC cell lines (Fig. 4G, H, S4D). It has been proved that high c-MYC expressing cells are more sensitive to Dasatinib [27–29]. We previously showed that by targeting YAP and TAZ, Dasatinib impairs SPAG5 expression in TNBC cells lines [16]. Indeed, 48 h of Dasatinib treatment, significantly reduced SPAG5 expression, as well as NME1 G3BP1, XRCC6, CCT3, TCP1 transcripts, in the three TNBC cell lines (Figs. 4A, C, D, F, S4A, S4C).

ChIP assay demonstrated that JQ-1 and Dasatinib reduced the recruitment of MYC-p(Ser62) and Histone H4 acetylation to the MYC-binding sites on SPAG5 promoter (Figs. 4I, JS4E, F).

JQ-1 treatment reduced the level mut-p53 and MYC-p(Ser62) proteins in MDA-MB 231 cell line (Fig. 4K, L), but, consistent with previous reports [30], little affect YAP protein and its nuclear localization (Fig. 4K, L); instead, significantly impaired YAP transcriptional activity, as shown by the reduced expression of SPAG5, CTGF, ANKRD1 and CYR61 (Figs. 4K, L, S4G–I) [26, 30, 31]. Dasatinib, weakened MYC-p(Ser62) and mut-p53 proteins expression, preserved YAP nuclear localization and caused to downregulation of CYR61, CTGF and ANKRD1 expression (Figs. 4K, L, S4G–I). Increase of p21 CDK inhibitor and poly (ADP-ribose) polymerase-1 (PARP-1) proteins was associated with the corresponding anti-proliferative and pro-apoptotic activity of JQ-1 and Dasatinib in TNBC cell line. These results together with the reduced expression of SPAG5, MYC-p(Ser62) and mut-p53 strongly suggest that Dasatinib and JQ-1 treatments directly target the oncogenic network of SPAG5-MYC-mut-p53 in TNBC cells (Fig. 4L).

### Anthracyclines and taxanes have no effect on SPAG5 expression in TNBC

**Fig. 5:**
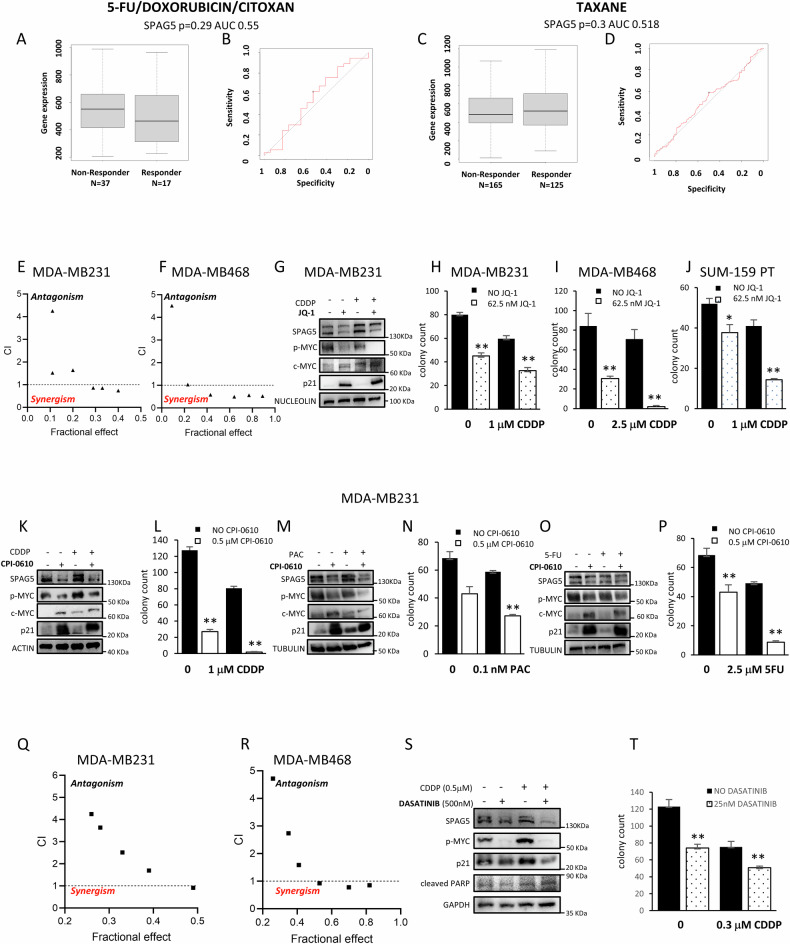
BET inhibitors and DASATINIB sensitized TNBC cell lines to chemotherapy. **A–C** Box-plot of SPAG5 expression in Responder and Non-Responder TNBC patients to 5-FU/DOXORUBICIN/CITOXAN (A) or TAXANE (C) treatments. **B–D** Roc Curves of SPAG5 expression in predicting Responder and Non-Responder TNBC patients to 5-FU/DOXORUBICIN/CITOXAN (B) or TAXANE (D) treatments. **E**, **F** Viability Assay. Synergy between JQ-1 and cisplatin was evaluated by cell viability assay using the ATPlite kit in MDA-MB231 (E) and in MDA-MB 468 (F) cells. **G** Western blot analysis of SPAG5, MYC-p(Ser62), c-MYC, and p21 protein levels in whole-cell lysate of MDA-MB231 cells, after treatment for 72 hr with 125 nM JQ-1 or 1 μM CDDP. Clonogenic assay. Representative micrographs of colonies formed by MDA-MB-231 (**H**), MDA-MB468 (**I**), and SUM-159 (**J**) cell lines pre-treated for 72 hr with 62.5 nM of JQ-1, and 1 μM of cisplatin before seeding at clonal density. After 7–10 days later, colonies were stained with crystal violet and counted. Column graphs show colony count and *P* value from three independent experiments. Western Blot analysis of SPAG5, MYC-p(Ser62), c-MYC, and p21 protein levels in whole-cell lysate of MDA-MB-231 cell line after treatment for 72 hr with 1 μM of CPI-0610, and 1 μM of cisplatin (**K**), 0.1 nM Paclitaxel (**M**) and 2.5 μM of 5-Fluorouracil (**O**). Clonogenic assay. Representative micrographs of colonies formed by MDA-MB-231 cell line pre-treated for 72 h with 0.5 μM of CPI-0610 and 1 μM of cisplatin (**L**) 0.1 nM Paclitaxel (**N**) and 2.5 μM of 5-Fluorouracil (**P**), before seeding at clonal density. After 7–10 days later, colonies were stained with crystal violet and counted. Column graphs show colony count and *P* value from three independent experiments. **Q**, **R** Viability assay. Dasatinib synergizes with cisplatin in MDA-MB231 (**Q**) and MDA-MB 468 (**R**) cells as evaluated by cell viability assay using the ATPlite kit. **S** Western blot analysis of SPAG5, MYC-p(Ser62), p21 and cleaved PARP protein levels in MDA-MB-231 cells following treatment with 500 nM DASATINIB and 0.5 μM of cisplatin. **T** Clonogenic assay. Representative micrographs of colonies of MDA-MB-231 cells pre-treated for 48 h with 25 nM DASATINIB and 0.3 μM cisplatin before seeding at clonal density. After 7–10 days later, colonies were stained with crystal violet and counted. Column graphs show colony count and *P* value from three independent experiments. (*P value < 0.05; **P value < 0.001).

Currently, standard care for TNBC include non-specific chemotherapy such as taxanes or anthracyclines. To investigate the clinical efficacy of SPAG5 to predict TNBC patient’s response to cytotoxic therapy we used the Rocplot analysis (www.rocplot.org) [32]. Non-significant modulation of SPAG5 expression was obtained between responsive and non-responsive cohorts in combination therapy of 5-Fluorouracil (5-FU) plus Cytoxan, Doxorubicin (p = 0.29; AUC = 0.55) (Fig. 5A) or Taxane (p = 0.3; AUC = 0.518) (Fig. 5C). Moreover, ROC curve analysis reveals non-predictive value of SPAG5 to establish patient’s response to combination therapy of 5-Fluorouracil plus Cytoxan and Adriamycin (Fig. 5B) or Taxane (Fig. 5D). Accordingly, sub-apoptotic doses of cisplatin (CDDP), 5-Fluorouracil, Doxorubicin, Paclitaxel and Vinorelbine didn’t affect SPAG5 protein expression in MDA-MB-231 and MDA-MB-468 TNBC cancer cell lines (Fig. S5A, B).

### BET-inhibitor sensitizes TNBC cell to chemotherapy

SPAG5 knockdown sensitized TNBC and cervical cancer cell lines to Olaparib and Taxol [9, 33]. Therefore, we tested whether JQ-1 treatment, which reduces SPAG5 expression, synergies with cisplatin to impair proliferation of TNBC cells.

Indeed, we found that pretreatment with JQ-1 sensitized MDA-MB231 (Table 2A) (Fig. S5C) and MDA-MB468 (Table 2B) (Fig. S5D) cells to cisplatin treatment (Figs. 5E, F, S5C, D). As shown in Fig. 5G, treatment of MDA-MB-231 cells with cisplatin weakly reduced MYC-p(Ser62) protein level. Intriguingly, compared with the single treatments, combination of JQ-1 and cisplatin led to a significant decrease of MYC-p(Ser62) and SPAG5 proteins in MDA-MB-231 cells (Fig. 5G). Likewise, combined treatment of JQ-1 with other cytotoxic based chemotherapy, 5-Fluorouracil, or Paclitaxel, significantly reduced MYC-p(Ser62) and SPAG5 expression in MDA-MB231 cells (Fig. S5H, I), and significantly increased the expression of CDK-inhibitor p21 (Fig. 5G, H, I).

**Table 2:** Combination Index (CI) of MDA-MB-231 (**A**) and MDA-MB-468 (**B**) cells treated with JQ-1 (250 nM) and different doses of cisplatin for 72 h. Combination Index (CI) of MDA-MB-231 (**C**) and MDA-MB-468 (**D**) cells treated with Dasatinib (0.5 μM) and different doses of cisplatin for 72 h.

A: MDA-MB-231		
JQ-1 (nM)	CDDP (μM)	Combination Index
250	0.25	4.250
250	0.5	1.522
250	1	1.640
250	2	0.862
250	4	0.854
250	8	0.741
**B: MDA-MB-468**		
**JQ-1 (nM)**	**CDDP (μM)**	**Combination Index**
250	0.25	4.515
250	0.5	1.031
250	1	0.581
250	2	0.493
250	4	0.559
250	8	0.523
**C: MDA-MB-231**		
**DASATINIB (μM)**	**CDDP (μM)**	**Combination Index**
0.5	0.25	4.247
0.5	0.5	3.637
0.5	1	2.512
0.5	2	1.693
0.5	4	0.911
0.5	8	0.898
**D: MDA-MB-468**		
**DASATINIB (μM)**	**CDDP (μM)**	**Combination Index**
0.5	0.25	4.7186
0.5	0.5	2.737
0.5	1	1.585
0.5	2	0.922
0.5	4	0.775
0.5	8	0.854

Combination Index was calculated by calcusyn software according to the manufacturer’s instructions.

Cisplatin, 5-Fluorouracil, and Paclitaxel slightly inhibited colony formation of TNBC cells, when administer as a single agent (Figs. 5H–J, S5E–G, S5J). However, combination of cisplatin, paclitaxel, or 5-Fluorouracil with JQ-1 farther reduce clonogenic ability of MDA MB-231, compared with single agent treatments (Fig. 5H, S5J). These results suggest that the synergistic effect of these compounds is partially mediated by impairment of MYC activity, and consequently of SPAG5 expression.

Next, we examined whether transient ectopic overexpression of SPAG5 could abolish the synergistic effect of JQ-1, cisplatin, paclitaxel, and 5-Fluorouracil in MDA-MB-231 cell line. As shown in Fig. S5K, MDA-MB-231 cells overexpressing SPAG5, gained resistance to JQ-1 treatment, and lost the synergy of JQ-1 with cisplatin, paclitaxel, or 5-Fluorouracil as shown by colony formation assay (Fig. S5K). These results suggest that SPAG5 is a leading player in the synergism between JQ-1 and cytotoxic based chemotherapy. To further corroborate these findings, we used more advanced BET inhibitors, which have been recently used in clinical trials, including CPI-0610 *(NCT02158858*, *NCT02157636)*, INCB054329 *(NCT02431260)* and OTX015 (*NCT02259114)*. As shown (Fig. S5L, M), treatment of MDA-MB-231 and MDA-MB-468 TNBC cell lines with sub apoptotic doses of CPI-0610, INCB054329, and OTX015, reduced MYC-p(Ser62) and SPAG5 proteins levels in a dose dependent manner. Concurrently, CDK-inhibitor p21 was increased, highlighting the anti-proliferative effects of these compounds. Moreover, combination treatment of CPI-0610 and cisplatin, paclitaxel, or 5-Fluorouracil markedly reduced MYC-p(Ser62) and SPAG5 proteins level, and significantly inhibited clonogenic ability of MDA-MB-231 cell line, compared to single agent treatments (Fig. 5K–P). All together, these results demonstrated that BET-inhibitors reduced SPAG5 expression via MYC, and sensitized TNBC cells to conventional therapy.

### Dasatinib compound synergies with cytotoxic chemotherapy in inhibiting SPAG5 expression and TNBC cell clonogenicity

A recent phase II clinical trial documented the safety of combined treatment of Dasatinib, plus Trastuzumab and Paclitaxel in HER-2-positive metastatic BC patients with acquired resistance to Trastuzumab (NCT01306942, EudraCT 2010-023304-27) [34].

To determine whether Dasatinib can synergize with cisplatin to inhibit SPAG5 expression and TNBC cell proliferation, we calculated their combination index in MDA-MB-231 and MDA-MB-468 cell lines (Fig. 5Q, R). As shown, pretreatment with Dasatinib sensitized MDA-MB-231 (Table 2C) and MDA-MB-468 (Table 2D) to cisplatin treatment (Fig. 5Q, R). As shown in Fig. 5S, cisplatin or Dasatinib treatment alone, had no or little effect, respectively, on SPAG5 expression but their combination markedly reduced SPAG5 level in MDA-MB-231 cells. Furthermore, a significant reduction of MDA-MB-231 colonies were obtained in response to the combined treatments (Fig. 5T). Collectively, these findings suggest that in preclinical models, undirected pharmacological targeting of MYC-SPAG5 axis perturb essential oncogenic features of TNBC.

### Targeting of SPAG5 expression reduces viability of patients derived tumor organoid (PDO)

**Fig. 6:**
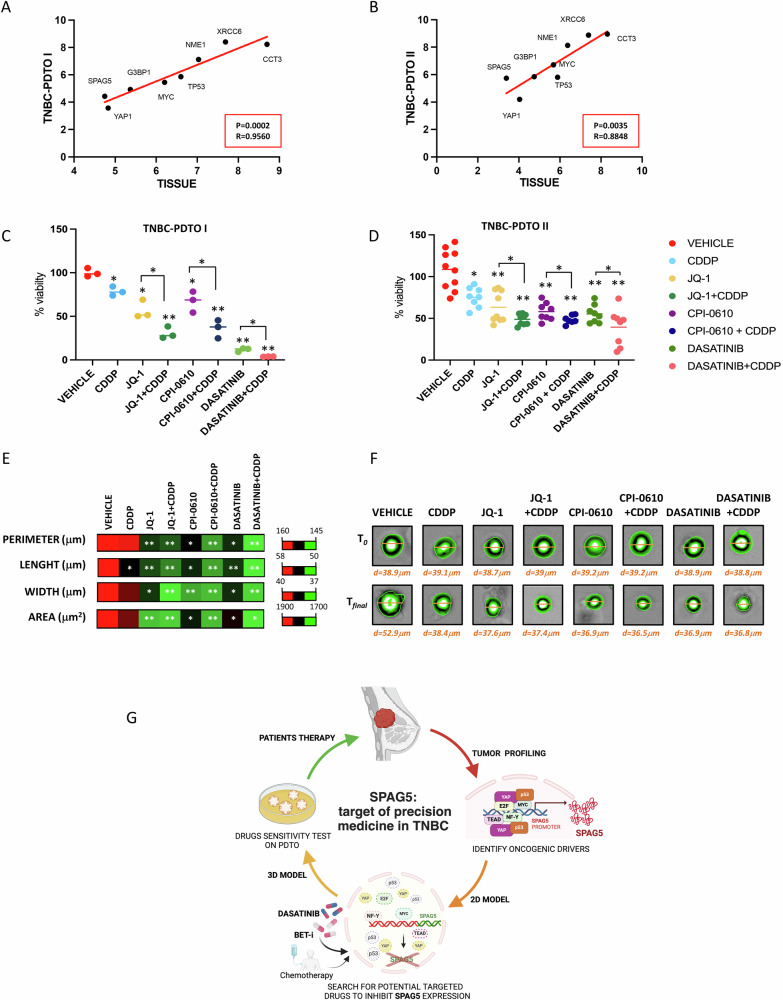
Targeting of SPAG5 reduces viability of TNBC patients derived tumor organoids (TNBC-PDTOs). **A**, **B** Pearson Correlation Analysis between expression value of MYC, G3BP1, XRCC6, CCT3, NME1, YAP1, SPAG5, TP53 genes in TNBC tissues and TNBC-PDTOs evaluated by RNA-seq analysis. **C**, **D** Viability assay cell viability of TNBC-PDTOs following cisplatin (1 μM), JQ-1 (62.5 nM), JQ-1 (62.5 nM) + cisplatin (1 μM), CPI-0610 (0.5 μM), CPI-0610 (0.5 μM) + cisplatin (1 μM), Dasatinib (0.3 μM) and Dasatinib (0.3 μM) + cisplatin (1 μM) treatments, evaluated by ATPlite assay. Graphs show cell viability and *P* value from three independent experiments. **E** Heat-maps display the perimeter, length, width, and area of TNBC-PDTO II after treatments with cisplatin (1 μM), JQ-1 (62.5 nM), JQ-1 (62.5 nM) + cisplatin (1 μM), CPI-0610 (0.5 μM), CPI-0610 (0.5 μM) + cisplatin (1 μM), Dasatinib (0.3 μM) and Dasatinib (0.3 μM) + cisplatin (1 μM). Data show the mean morphological measurement and p-value from 8 technical experiments on TNBC-PDTO II assessed by Opera Phenix. **F** Representative images of TNBC-PDTO II following treatments with cisplatin (1 μM), JQ-1 (62.5 nM), JQ-1 (62.5 nM) + cisplatin (1 μM), CPI-0610 (0.5 μM), CPI-0610 (0.5 μM) + cisplatin (1 μM), Dasatinib (0.3 μM), and Dasatinib (0.3 μM) + cisplatin (1 μM). d = diameter (μm) assessed by Opera Phenix. p-value was calculated over non treated samples (*P value < 0.05; **P value < 0.001). **G** Proposed model.

To translate our findings on three-dimensional (3D) system we used TNBC patients derived tumor organoids (TNBC-PDTOs) [35–37]. RNA-seq analysis reveals similar expression levels of SPAG5, MYC, TP53, YAP1, G3BP1, XRCC6, CCT3, NME1 genes between parental tumoral tissues and TNBC-PDTOs (p = 0.0002, R = 0.9560 and p = 0.0035 and R = 0.8848) (Fig. 6A, B Table 3).

**Table 3:** Log2 CPM expression value of MYC, G3BP1, XRCC6, CCT3, NME1, YAP1, SPAG5, TP53 genes in TNBC tissues and TNBC-PDTOs.

	MYC	G3BP1	XRCC6	CCT3	NME1	YAP1	SPAG5	TP53
Tissue I	6.20288	5.37046	7.68701	8.69760	7.03328	4.74609	4.83039	6.60106
PDTO I	5.45695	4.92933	8.40356	8.22972	7.12291	4.42634	3.57182	5.85845
Tissue II	5.684215	4.7482	7.39272	8.30929	6.37506	4.03658	3.39409	5.88334
PDTO II	6.71540	5.84416	8.87511	8.95031	8.13082	4.19322	5.73350	5.80259

Indeed, we analyzed organoids viability following JQ-1, CPI-0610 or Dasatinib treatments, alone or in combination with cisplatin. As shown in Fig. 6C, D when administrated as a single agent treatment, cisplatin slightly reduced viability of TNBC-PDTO. JQ-1 treatment inhibited TNBC-PDTO proliferation by almost 30%, while combination of JQ-1 with cisplatin inhibited TNBC-PDTO viability by almost 60%, highlighting the potency of this combination. We also tested the efficacy of CPI-0610. CPI-0610 alone, reduced the viability of TNBC-PDTO, but more profoundly together with cisplatin (Fig. 6B, D). Dasatinib had strong effect on PDTO viability as a single drug, and even stronger effect in combination with cisplatin (Fig. 6B, D). Therapeutic interventions can modify organoid morphology. To assess the efficacy of drug treatments on TNBC organoids, we measured perimeter, length, width, and area. As shown in Fig. 6E, F CDDP treatment resulted in minimal changes in these parameters, whereas JQ1, Dasatinib and CPI-0610 had more pronounced effects on organoid architecture. Furthermore, the combination of JQ1, CPI-0610 and Dasatinib with cisplatin led to more significantly reduced the perimeter, length, width, and organoids area, corroborating the viability data (Fig. 6E, F). Altogether, our results, marked SPAG5 expression as a bona fide biomarker to identify patient’s responder and/or non responders to MYC inhibitors and their combination with chemotherapy.

## Discussion

TNBCs are high invasive and proliferative tumors, which hide extensive inter- and intra-tumoral heterogeneity [2, 38]. Due to the lack of effective target, the current therapeutic approach for TNBC is cytotoxic chemotherapy, but acquired resistance and relapse are hurdle still difficult to overcome [39]. In our study, we used gene expression profiling and mechanistic assays to uncover a complex oncogenic network regulating SPAG5 expression in TNBC.

SPAG5 promotes proliferation and chemoresistance, inhibits apoptosis and its overexpression is associated with poor prognosis in many different tumors [10, 40]. Frequently high expressed in recurrent and metastatic BCs, in TNBC, elevated SPAG5 expression was associated with high levels of Ki67, TOP2A, BIRC5, AURKA, BUB1, and ATR-BRCA pathways [8, 9, 41, 42]. The large expression of cell cycle related genes increased immune tumoral infiltration; the positive association between cancer’s antigens and infiltrating immune cells is one of the suggested criteria applied to develop effective anti-cancer vaccine. Interestingly, SPAG5 overexpression was correlated with high levels of lymphocyte CD8+, macrophages, neutrophils, dendritic and B cells, positively associated with PD-1/PDL1, LAG3, GZMB and CTLA4 and SPAG5 antigens were suggested as valid immune stimulatory targets for anti-cancers therapy [40, 43, 44].

Over 80% of TNBC express mut-p53 protein [45]. Previous data links SPAG5 overexpression with p53 signaling in HCC and lung cancer [40, 46]. Here, we demonstrated that in BC cell lines depletion of mut-p53 reduce SPAG5 transcripts and proteins expression. Moreover, mut-p53 directly recruits transcription factors on SPAG5 promoter. Accordingly, targeting of mut-p53 impairs c-MYC activity and SPAG5 expression. It has been reported that in TNBC, by interacting with MYC-BP, SPAG5 promotes MYC transcriptional activation [9]. Here we demonstrated that SPAG5 is a direct transcriptional target of MYC, raising the possibility that BC progression was partially sustained by a MYC-SPAG5 positive feedback loop.

Some pre-clinical studies, evidenced antitumoral activity and MYC sensitivity to BET-inhibitors and Dasatinib; nevertheless, their limited clinical application was bounded by the lacks of specific and sensitive biomarkers [47]. We show that in MDA-MB-231 TNBC cell line, JQ-1 and Dasatinib directly impaired MYC-p(Ser62) recruitment and Histone H4 acetylation on SPAG5 promoter, reduced SPAG5 expression and oncogenic proprieties of TNBC cell lines. We tested SPAG5 expression as an effective predictive biomarker for advanced BET-inhibitors compounds: CPI-0610, INCB054329 and OTX015 treatments, markedly reduced MYC-p(Ser62) activity and SPAG5 expression, leading TNBC cells to growth arrest.

One of the limitations in the application of BET-inhibitors in clinical setting, is the deriving intrinsic resistance over multiple cancers, when used as a single agent [47]. To overcome this concern, we tested the synergism between JQ-1 and cytotoxic drugs, cisplatin, paclitaxel, or 5-Fluorouracil. Importantly, whereas Alkylating drugs and Taxane didn’t affect SPAG5 expression, combined treatments of JQ-1 or CPI-0610 with cisplatin, paclitaxel, or 5-Fluorouracil, decreased MYC-p(Ser62) and SPAG5 expression, and markedly reduced proliferation of TNBC cell lines.

We demonstrate that by undirected targeting of MYC-SPAG5 axis, we sensitize TNBC-PDTOs to cisplatin treatment, eliciting reliable inhibition of cell viability. Nowadays, effective therapeutic model for drugs testing is one of the major challenges in the oncologic fields [37, 48, 49]. Our TNBC-PDTO models, displays similar drugs response to TNBC cell lines, corroborating the relevance of our results and the potential of their clinical application.

Our translational approach provides strong evidence that genetic profiling of TNBC should improve selection of potential actionable targets, that is one of the primary issues in the development of advanced precision medicine. Along with chemotherapy, novel anticancer strategies are mandatory to improve TNBC patient’s prognosis, and, in this context, SPAG5 overexpression should be used as a predictive biomarker for effective anticancer therapies (Fig. 6C).

### Supplementary information

Supplementary File

Original Western Blot

## Data Availability

The datasets generated during and/or analyzed during the current study are available from the corresponding author on reasonable request.
