# Supplementary material for: Targeting of mutant-p53 and MYC as a novel strategy to inhibit oncogenic SPAG5 activity in triple negative breast cancer
Source: Cell Death Dis. 2024 Aug 20;15(8):603. doi: 10.1038/s41419-024-06987-x (PMC11336084; doi:10.1038/s41419-024-06987-x)
Supplement: Supplementary file 1 — Supplementary File [file 41419_2024_6987_MOESM1_ESM.pdf]

Supplementary Figures

Suppl Fig1

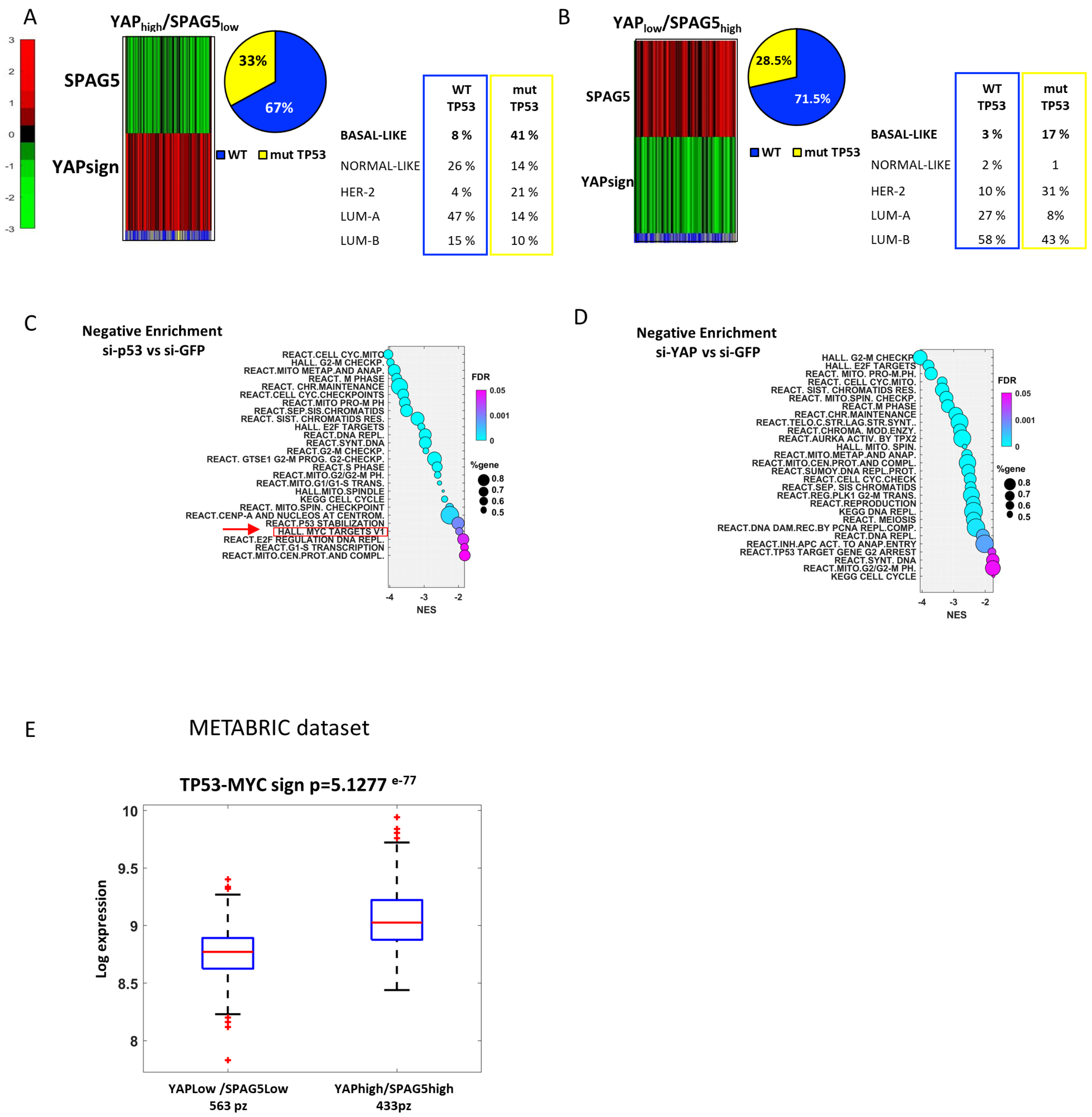

Supplementary Fig. 1: (A, B) Heatmaps (on the left) of normalized expression levels of SPAG5, YAP and p53 status in METABRIC dataset. **Pie-charts** (in the middle) represent frequencies of p53

mutation in breast cancer patients from METABRIC dataset stratified for combined expression of SPAG5 and YAP. **Tables** (on the right) breast cancer histotype classification in the different group of breast cancer patients stratified for combined expression of TP53, SPAG5 and YAP. (**C, D) Dot plots** of the gene sets obtained by pre-ranked GSEA on MDA-MB-468 breast cancer cell 48 hr post transfection with sip53 (C) or siYAP (D). (**E) Box-plot** of TP53-MYC-target genes signature expression in breast cancer patients from METABRIC dataset, stratified for different combined levels of SPAG5 and YAP signature expression.

Suppl Fig 2

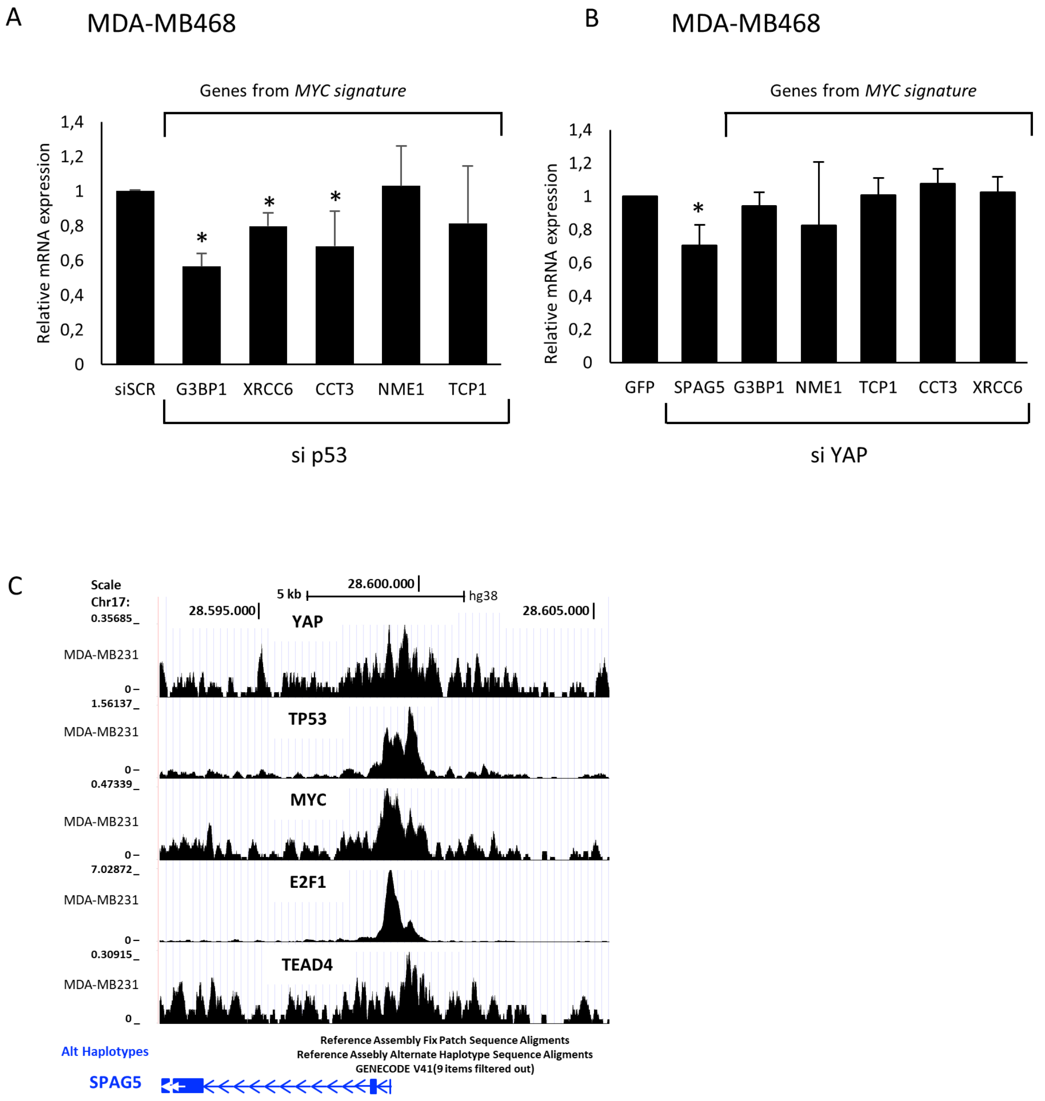

Supplementary Fig. 2: (A, B) Expression level of SPAG5, G3BP1, CCT3, XRCC6, TCP1 and NME1 in MDA-MB-468 was assessed by quantitative PCR 48 hr post transfection with sip53 (A) and siYAP (B) siRNA transfection. (**C) ChIP-seq Peaks** of YAP, TP53, MYC, E2F-1 and TEAD4

on SPAG5 promoter as assessed by *cistrome-GO* databrowser (http://go.cistrome.org). (*P value <0.05; **P value <0.001).

Suppl Fig 3

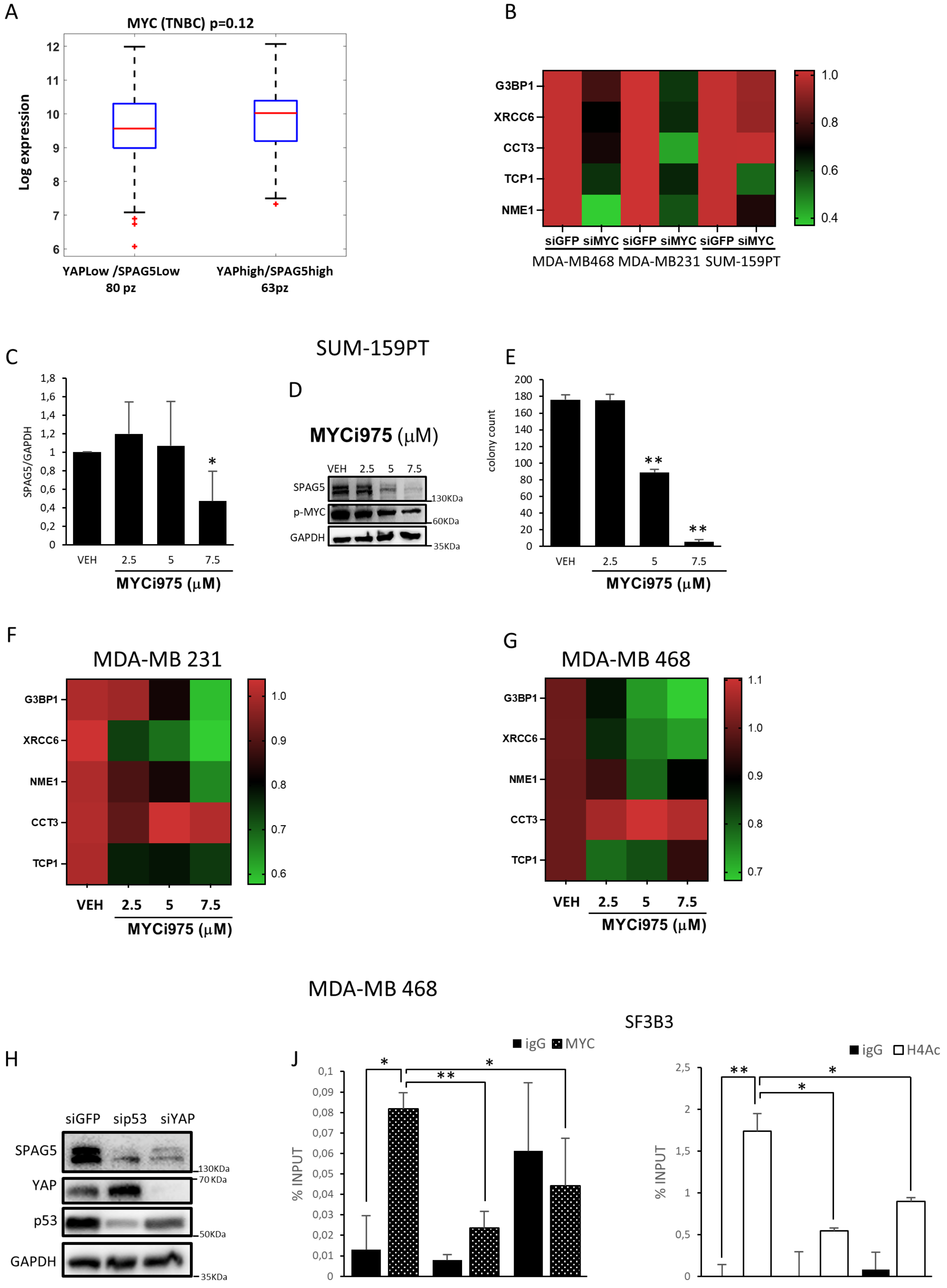
A
MYC (TNBC) p=0.12
Log expression
12
11
10
9
8
7
6
YAPLow /SPAG5Low
80 pz
YAPhigh/SPAG5high
63pz
B
G3BP1
XRCC6
CCT3
TCP1
NME1
1.0
0.8
0.6
0.4
siGFP siMYC siGFP siMYC siGFP siMYC
MDA-MB468 MDA-MB231 SUM-159PT
SUM-159PT
C
SPAG5/GAPDH
1,8
1,6
1,4
1,2
1
0,8
0,6
0,4
0,2
0
*
VEH
2.5
5
7.5
MYCi975 (μM)
D
MYCi975 (μM)
VEH 2.5 5 7.5
SPAG5
p-MYC
GAPDH
130KDa
60KDa
35KDa
E
colony count
200
180
160
140
120
100
80
60
40
20
0
**
**
VEH
2.5
5
7.5
MYCi975 (μM)
F
MDA-MB 231
G3BP1
XRCC6
NME1
CCT3
TCP1
1.0
0.9
0.8
0.7
0.6
VEH 2.5 5 7.5
MYCi975 (μM)
G
MDA-MB 468
G3BP1
XRCC6
NME1
CCT3
TCP1
1.1
1.0
0.9
0.8
0.7
VEH 2.5 5 7.5
MYCi975 (μM)
MDA-MB 468
SF3B3
H
siGFP sip53 siYAP
SPAG5
YAP
p53
GAPDH
130KDa
70 KDa
50KDa
35KDa
J
igG MYC
% INPUT
0,1
0,09
0,08
0,07
0,06
0,05
0,04
0,03
0,02
0,01
0
*
**
*
siSCR
sip53
siYAP
igG H4Ac
% INPUT
2,5
2
1,5
1
0,5
0
**
*
*
siSCR
sip53
siYAP

Supplementary Fig. 3: (A) Box-plots expression levels of c-MYC in TNBC patients from METABRIC dataset, stratified for different combined levels of SPAG5 and YAP signature expression. (**B) Heatmap** shows the expression levels of G3BP1, CCT3, XRCC6, TCP1 and NME1 in MDA-MB-468, MDA-MB-231 and SUM-159 TNBC cell lines as assessed by qPCR 48 hr after siGFP or siMYC siRNA transfection. **(C) qPCR** Expression levels of SPAG5 in SUM-159 was assessed by quantitative PCR 48 hr after treatment with 2.5µM -5µM -7.5µM of MYCi975. (**D) Western blot** analysis of SPAG5 and p-MYC protein levels in whole-cell lysate of SUM-159, 48 hr post treatment with 2.5µM -5µM -7.5µM of MYCi975. **(E) Clonogenic assay.** Representative micrographs of colonies formed by SUM-159 treated for 48 hr with 2.5µM -5µM -7.5µM of MYCi975, before seeding at clonal density. After 7-10 days later, colonies were stained with crystal violet and counted. Column graphs show colony count and *P* value from three independent experiments. (**F-G) Heatmaps** show the expression levels of G3BP1, CCT3, XRCC6, TCP1 and NME1 in MDA-MB-231 (H) and MDA-MB-468 (I) TNBC cell lines, as assessed by qPCR, 48 hr post treatment with 2.5µM -5µM -7.5µM of MYCi975. (**H) Western blot** analysis of SPAG5, YAP, and p53 protein levels in whole-cell lysate of MDA-MB-468 cells 48 hr post transfection with siGFP, siYAP, and sip53 siRNAs. **(J) ChIP analysis** of SF3B3 promoter in MDA-MB-468 cells after knocking down YAP or p53. (*P value <0.05; **P value <0.001).

Suppl Fig 4

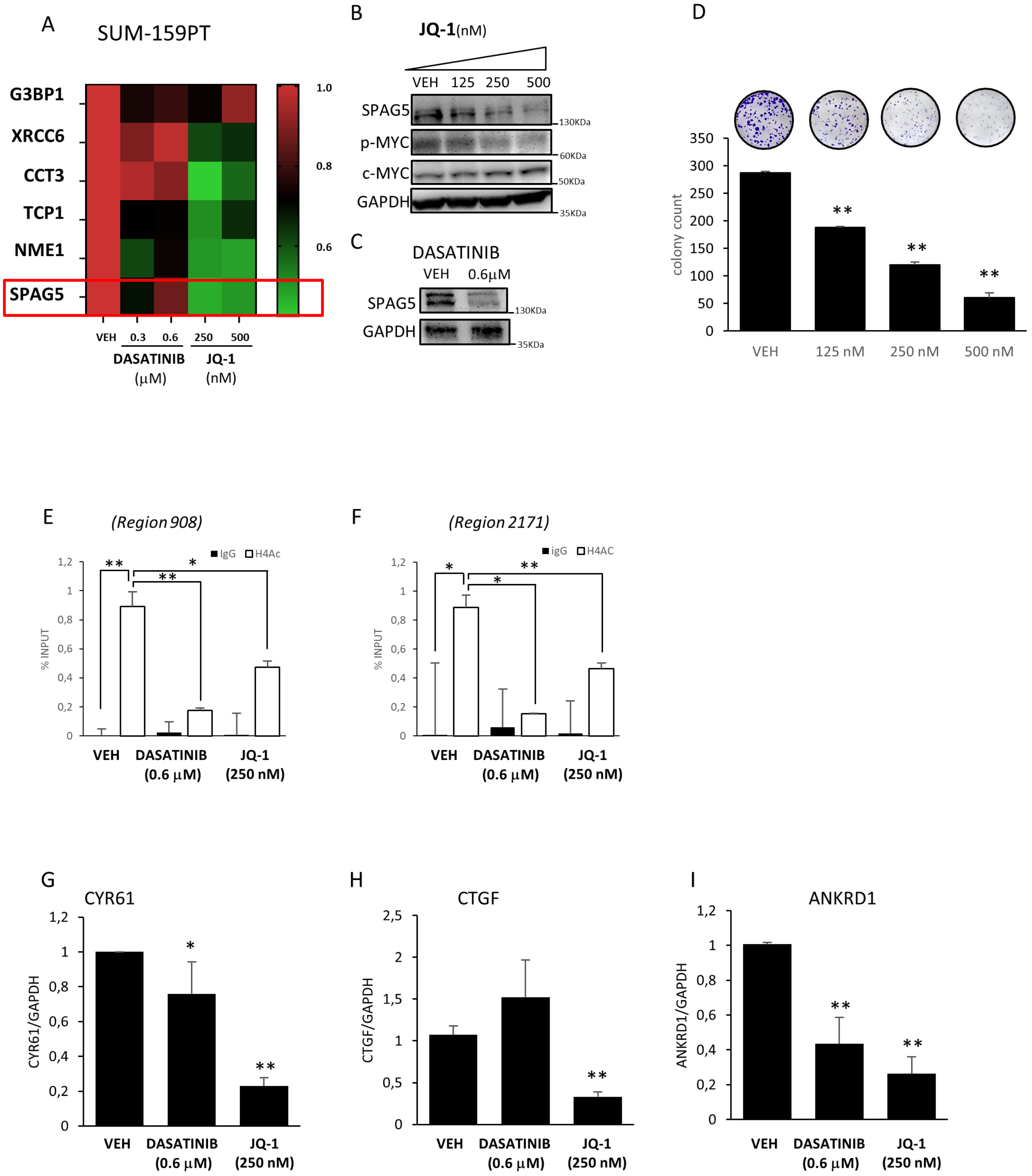

Supplementary Fig. 4: (A) Heatmap shows the expression levels of SPAG5, G3BP1, XRCC6, CCT3, TCP1 and NME1 in SUM-159 TNBC cell line 48 hr post treatment with 0.3-0.6 µM DASATINIB and 72 hr with 250-500 nM JQ-1 treatments. **(B, C) Western blot** analysis of SPAG5,

MYC-p(Ser62), and c-MYC protein levels in whole-cell lysate of SUM-159 cells 72 hr post treatment with 125-250-500 nM JQ-1 (**B**), or 48 hr with 0.6 μM DASATINIB (**C**). (**D) Clonogenic assay.** Representative micrographs of colonies formed by SUM-159 cells treated for 72 hr with 125-250-500 nM JQ-1 before seeding at clonal density. After 7-10 days later, colonies were stained with crystal violet and counted. Column graphs show colony count and *P* value from three independent experiments. (*P value <0.05; **P value <0.001).

Suppl Fig 5

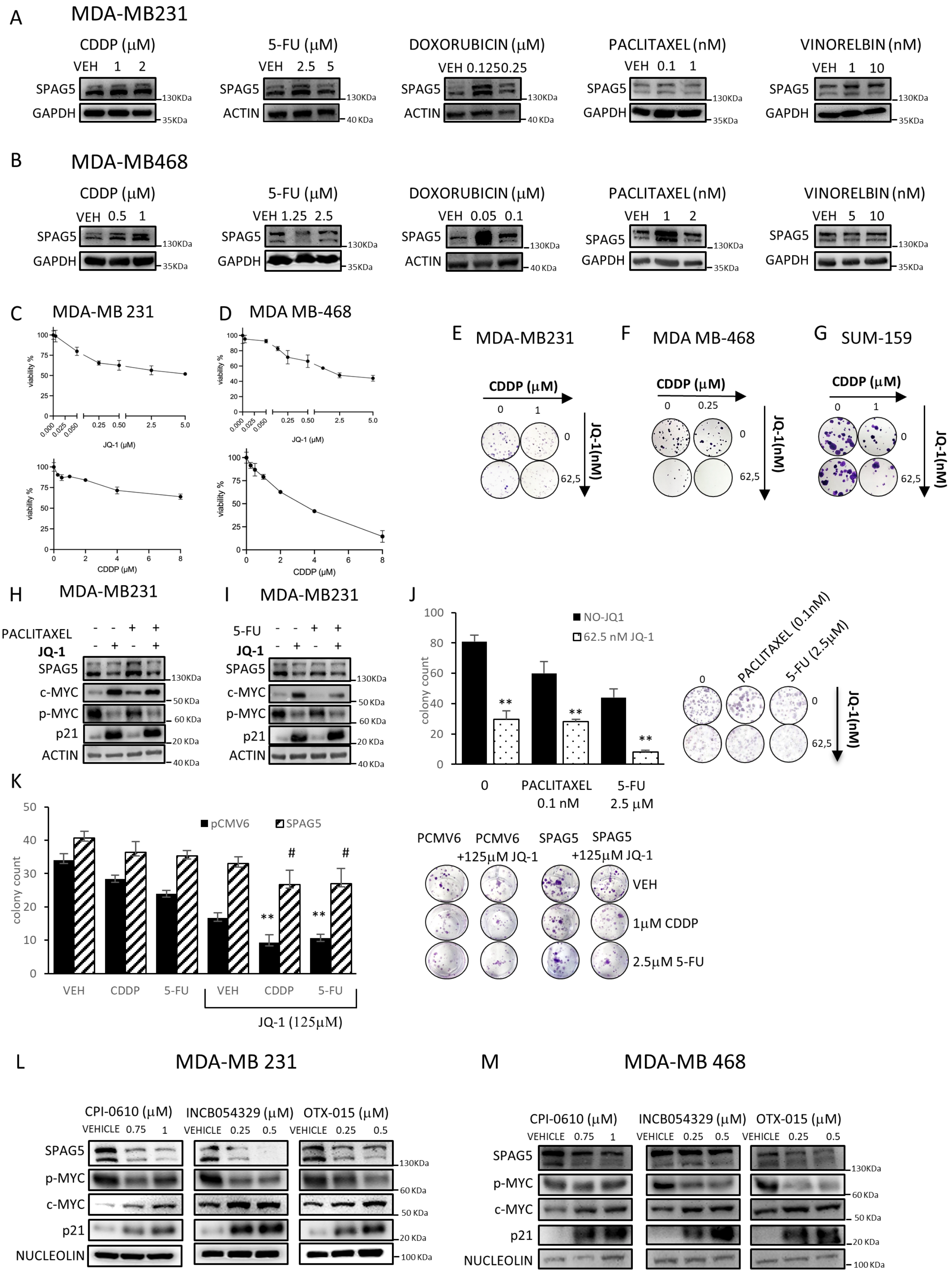
A
MDA-MB231
CDDP (μM)
VEH 1 2
SPAG5
GAPDH
5-FU (μM)
VEH 2.5 5
ACTIN
DOXORUBICIN (μM)
VEH 0.125 0.25
PACLITAXEL (nM)
VEH 0.1 1
VINORELBIN (nM)
VEH 1 10
130KDa
35KDa
40 KDa
B
MDA-MB468
CDDP (μM)
VEH 0.5 1
5-FU (μM)
VEH 1.25 2.5
DOXORUBICIN (μM)
VEH 0.05 0.1
PACLITAXEL (nM)
VEH 1 2
VINORELBIN (nM)
VEH 5 10
C
MDA-MB 231
D
MDA MB-468
viability %
JQ-1 (μM)
CDDP (μM)
E
MDA-MB231
F
MDA MB-468
G
SUM-159
CDDP (μM)
JQ-1(nM)
62,5
H
MDA-MB231
PACLITAXEL
JQ-1
I
MDA-MB231
5-FU
SPAG5
c-MYC
p-MYC
p21
ACTIN
50 KDa
60 KDa
20 KDa
J
NO-JQ1
62.5 nM JQ-1
colony count
0
PACLITAXEL 0.1 nM
5-FU 2.5 μM
PACLITAXEL (0.1nM)
5-FU (2.5μM)
K
pCMV6
SPAG5
VEH
CDDP
5-FU
JQ-1 (125μM)
PCMV6
PCMV6 +125μM JQ-1
SPAG5
SPAG5 +125μM JQ-1
VEH
1μM CDDP
2.5μM 5-FU
L
MDA-MB 231
M
MDA-MB 468
CPI-0610 (μM)
INCB054329 (μM)
OTX-015 (μM)
VEHICLE 0.75 1
VEHICLE 0.25 0.5
SPAG5
p-MYC
c-MYC
p21
NUCLEOLIN
100 KDa

Supplementary Fig. 5: (A, B) Western blot analysis of SPAG5 protein levels in whole-cell lysate of MDA-MB-231 (A) or MDA-MB-468 (B) TNBC cell lines, treated for 72 hr with the specified chemotherapeutic compounds. (**C, D) Viability Assay** was used to evaluate the sensitivity to different JQ-1 and CDDP doses in MDA-MB231 (C) and MDA-MB-468 (D) cells using the ATPlite kit. Results are presented as percentage of cell viability relative to the untreated control cells. (**E-G)** Pictures of colony assay from MDA-MB-231 (E), MDA-MB-468 (F), and SUM-159 (G), TNBC cell lines treated with JQ-1 and cisplatin. **(H-I) Western blot** analysis of SPAG5, MYC-p(Ser62), c-MYC, and p21 protein levels in whole-cell lysate of MDA-MB-231 cells treated with 125 nM JQ-1 and 0.1 nM Paclitaxel (H), or 2.5 μM 5-Fluorouracil (I). (**J) Clonogenic assay.** Representative micrographs of colonies formed by MDA-MB-231 cells pre-treated for 72 hr with 62.5 nM JQ-1 and 0.1 nM Paclitaxel, or 2.5 μM of 5-Fluorouracil, before seeding at clonal density. After 7-10 days later, colonies were stained with crystal violet and counted. Column graphs show colony count and *P* value from three independent experiments. **(K) Clonogenic assay**. Representative micrographs of colonies formed by MDA-MB-231 cell line overexpressing either pCMV6 or SPAG5-expression vector, pre-treated for 72 hr with 125 nM JQ-1 and 1 μM of cisplatin or 2.5 μM 5-Fluorouracil. After 7-10 days later, colonies were stained with crystal violet and counted. Column graphs show the number of colonies and *P* value was calculated from three independent experiments. (**L,M) Western blot** showing SPAG5, MYC-p(Ser62), c-MYC and p21 protein levels in whole-cell lysate of MDA-MB-231 (L) and MDA-MB-468 (M) cells, 72 hr post treatment with CPI-0610, INCB54329, or OTX-015. (*P value <0.05; **P value <0.001).
